# Emergency endoscopic variceal ligation following variceal rupture in patients with advanced hepatocellular carcinoma and portal vein tumor thrombosis: a retrospective study

**DOI:** 10.1186/s12957-016-0802-z

**Published:** 2016-02-24

**Authors:** Toshihiro Kawai, Yoko Yashima, Takafumi Sugimoto, Takahisa Sato, Miho Kanda, Nobuyuki Enomoto, Shinpei Sato, Shuntaro Obi

**Affiliations:** First Department of Internal Medicine, Faculty of Medicine, University of Yamanashi, 1110 Shimokawato, Chuo, Yamanashi 409-3898 Japan; Gastroenterology and Hepatology, Kyoundo Hospital, Sasaki Institute, Tokyo, Japan

**Keywords:** Esophageal varices, Endoscopic variceal ligation, Hepatocellular carcinoma, Portal vein tumor thrombus

## Abstract

**Background:**

The outcomes of treatment of ruptured varices in patients with hepatocellular carcinoma (HCC) and portal vein tumor thrombus (PVTT) are unclear. We therefore evaluated the long- (rebleeding and death) and short-term (immediate death within 24 h of variceal bleeding diagnosis) outcomes of patients with PVTT who underwent emergency variceal band ligation.

**Methods:**

Data on 62 patients with PVTT and endoscopically proven esophageal or gastric variceal bleeding from 2007 to 2012 were studied. In most cases, the varices were treated using endoscopic variceal band ligation (EVL). We assessed the patients’ rebleeding-free and overall survival using the Kaplan-Meier method, and a Cox proportional hazard model was used to analyze effect of independent factors on rebleeding-free and overall survival times.

**Results:**

Most patients had decompensated cirrhosis and were classified as Child-Pugh class B (56 %) or C (36 %). A total of 35 patients (56 %) had PVTT in the main portal trunk. Among all patients, 58 (94 %) and 4 (6 %) had esophageal and gastric variceal bleeding, respectively. Bleeding was managed using EVL in all, but one patient (98 %) who was treated with a Sengstaken-Blakemore tube. A total of 24 patients (39.3 %) experienced rebleeding, and these patients had a median overall survival time of 36 days. A PVTT in the main portal trunk was predictive of rebleeding (hazard ratio 3.706, *p* = .0223), and α-fetoprotein-L3 levels <37.4 % (hazard ratio 0.464, *p* = 0.015) and Child-Pugh class A/B (hazard ratio 0.398, *p* = 0.007) were associated with overall survival. We observed 95 bleeding events in 62 patients. EVL achieved hemostasis in 92 of the 95 bleeding events, whereas seven immediate deaths occurred due to variceal bleeding (7/92, 7.6 %). All three bleeding events treated with modalities other than EVL resulted in immediate deaths.

**Conclusions:**

EVL is a safe and effective treatment of variceal ruptures in patients with HCC and PVTT. After successful hemostasis, alleviation of the underlying liver function impairment and tumor control are equally important for a good prognosis.

## Background

Hepatocellular carcinoma (HCC) is the sixth most common cancer worldwide [1]. Due to the condition’s very poor prognosis, the number of deaths due to HCC is similar to its incidence, making it the third most common cause of cancer-related deaths [1]. The majority of HCC cases develop as part of the natural course of liver cirrhosis [2]. Despite the recommended surveillance, HCC is often only detected at an advanced stage [3, 4], particularly in patients with a portal vein tumor thrombus (PVTT; 12.5–39.7 % of cases) [4–8]. Although an increasing variety of therapeutic options that offer survival benefits are available for patients with HCC [9], the long-term prognosis of HCC remains poor because HCC recurrence and PVTT development remain uncontrollable [10].

A PVTT significantly affects the prognosis of HCC patients because it often leads to the extensive spread of the tumor throughout the liver and increases portal blood pressure, resulting in variceal ruptures, ascites, hepatic encephalopathy, liver failure, and death [11]. There has been a substantial improvement in the survival of patients with variceal bleeding owing to the use of vasoactive drugs [12], the introduction of endoscopic variceal band ligation (EVL) [13–15] and endoscopic injection sclerotherapy (EIS) [16, 17]. Several studies have examined the prognoses associated with variceal bleeding, but most have been observational studies that have excluded patients with HCC [18–23]. Therefore, the long-term outcomes of ruptured variceal treatment in patients with advanced HCC and PVTT remain unclear.

This study evaluated the long- (rebleeding and death) and short-term (immediate death) outcomes of HCC patients with ruptured esophageal or gastric varices and a PVTT.

## Methods

### Study design, setting, and data collection

We conducted a retrospective study using data from a database that included all patients admitted to the Kyoundo Hospital (Tokyo, Japan) for HCC between January 2007 and December 2012. The database was part of a project to evaluate the effect of current therapies on the prognosis of PVTT-related variceal bleeding and was approved by the Institutional Review Board of the Kyoundo Hospital. All data were collected in the context of standard practice from patient clinical records, anonymized, and populated into a protected database.

### Diagnosis of hepatocellular carcinoma, portal vein tumor thrombi, and variceal bleeding

HCC was diagnosed based on standard computed tomography (CT) findings [24]. A PVTT was defined as the presence of HCC invading the main trunk or the first or second branches of the portal vein on CT findings. Gastric varices were classified based on the following criteria [25]: Gastroesophageal varices (GOVs) were associated with varices along the lesser curvature of the stomach (type 1 [GOV1]) or along the fundus (type 2 [GOV2]). Isolated gastric varices (IGVs) were present in isolation in the fundus, at ectopic sites in the stomach (IGV1), or at the first part of the duodenum (IGV2). The status of each patient was assessed at the time of admission.

All patients were diagnosed as having bleeding esophageal or gastric varices by emergency endoscopy within 24 h of the hemorrhage. Acute variceal bleeding was diagnosed when blood was observed to emanate from a varix, or when fresh blood was seen in the esophagus of patients with varices displaying the white nipple sign [26] and in whom no other potential site of bleeding was identified. Standard therapy for variceal bleeding included blood transfusions, fluid and electrolyte replacement, and lactulose administration, as necessary.

### Endoscopic variceal band ligation

Following the emergency endoscopy to identify the bleeding points, a flexible overtube (Sumitomo Bakelite, Tokyo, Japan) was inserted, and the esophageal varices were ligated using a pneumatic EVL device (Sumitomo Bakelite). Ligation was initially applied at the bleeding point and performed with 1–3 rubber bands. After the ligation was completed, the ligation sites were sprayed with water and suctioned to check for persisting bleeding. Rescue therapies (e.g., a Sengstaken-Blakemore balloon tamponade [27]) were applied when necessary. Initial success was defined as bleeding cessation and vital sign stabilization.

### Follow-up of patients

Follow-up of the enrolled patients was continued until December 2013. Data regarding patient demographics, liver disease, and bleeding episodes were registered in the database.

### Variables and statistical analysis

Recurrent bleeding was defined as hematemesis recurring after initial EVL treatment. Immediate death was defined as death within 24 h of diagnosing the variceal bleeding. Rebleeding-free survival was calculated from the initial date of the therapy to the date of rebleeding or death and assessed by the Kaplan-Meier method. Overall survival was defined as the interval from the initial treatment to death. A Cox proportional hazard model was used to analyze the effects of independent factors on survival. Possible predictors entered into the model included patient characteristics (age and sex), viral characteristics (hepatitis B virus surface antigen and hepatitis C virus antibody levels), the presence of ascites, the presence of encephalopathy, tumor characteristics (maximum tumor dimension, tumor number, portal vein invasion, and extrahepatic metastasis), laboratory test results (albumin, alanine transaminase, aspartate transaminase, and total bilirubin levels; prothrombin time; and platelet count), and serum levels of tumor markers (α-fetoprotein [AFP], AFP-L3, and des-gamma carboxyprothrombin [DCP]), site of variceal rupture, EVL success, and the presence of rebleeding varices. Each continuous variable was transformed into categorical data consisting of two ordinal numbers using the median value for Cox regression analyses, except for AFP and DCP. The cutoff values were set at 100 ng/mL and 100 mAU/mL for AFP and DCP, respectively [10]. All statistical analyses were considered significant at *p* < 0.05. Statistical analysis was performed with the Stat View 5.0 statistical software (SAS Institute, Cary, NC, USA).

## Results

### Patient characteristics

**Fig. 1:**
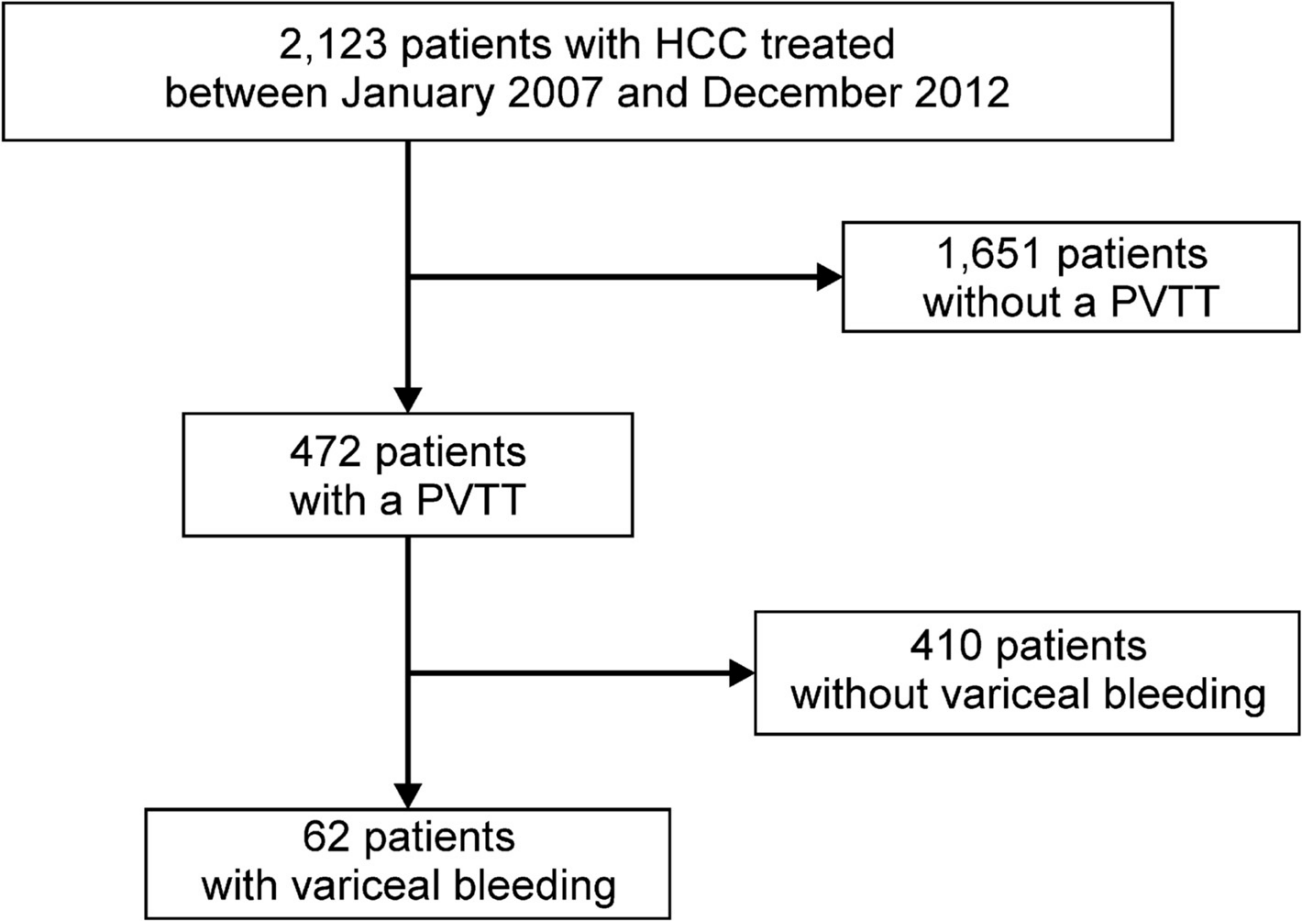
Flowchart of the study cohort selection. A total of 2123 hepatocellular carcinoma patients who were admitted to the Kyoundo Hospital between January 2007 and December 2012 were retrospectively registered in the database. Among these, 472 patients (22.2 %) had a portal vein tumor thrombus, and 62 (62/472; 13.1 %) experienced bleeding varices. *HCC* hepatocellular carcinoma, *PVTT* portal vein tumor thrombus

A total of 2123 HCC patients who were admitted to the Kyoundo Hospital between January 2007 and December 2012 were retrospectively registered in the database. Among these, 472 patients (22.2 %) had a PVTT and 62 (62/472; 13.1 %) were diagnosed with bleeding varices. Thus, the final analysis included 62 patients (Fig. 1).

The median age of the patients was 62 years (range, 30–79 years), 51 (82 %) were men and 11 (18 %) women (Table 1). The patients’ liver cirrhosis etiologies were hepatitis B (*n* = 25; 40 %), hepatitis C (*n* = 27; 44 %), and non-B non-C hepatitis (*n* = 10; 16 %). The patients were classified based on their liver function as Child-Pugh classes A (*n* = 5; 8 %), B (*n* = 35; 56 %), or C (*n* = 22; 36 %). Among all patients, 56 % had a PVTT in the main portal trunk, and 27 % had extrahepatic metastasis. None of the patients had a history of preventive procedures for varices.

**Table 1:** Patient baseline characteristics

Variable	
Age (years)	62 (30–79)
Sex (male/female)	51/11
Viral infection (HBV/HCV/NBNC)	25/27/10
Ascites (+/−)	12/50
Encephalopathy (+/−)	8/54
Albumin (g/dL)	2.9 (1.7–4.1)
Total bilirubin (mg/dL)	1.4 (0.4–21.4)
Prothrombin time (%)	68 (15–120)
Platelet count (× 10^4^/mm^3^)	12.0 (2.6–67.2)
AST (IU/L)	117.0 (26–2882)
ALT (IU/L)	48.5 (11–865)
Child-Pugh class (A/B/C)	5/35/22
Tumor size (cm)	6.8 (1.6–20)
Tumor number (single/multiple)	5/57
Extrahepatic metastasis (+/−)	17/45
PVTT (main trunk/others)	35/27
AFP (ng/dL)	1780.5 (2–3535473)
AFP-L3 (%)	37.4 (0.5–94.2)
DCP (ng/dL)	7260 (7–402000)
Variceal rupture (esophagus/stomach)	58/4

*AFP* α-fetoprotein, *AFP-L3* lectin-reactive α-fetoprotein, *ALT* aspartate aminotransferase, *AST* alanine aminotransferase, *DCP* des-gamma carboxyprothrombin, *HBV* hepatitis B virus, *HCV* hepatitis C virus, *NBNC* non-B non-C hepatitis, *PVTT* portal vein tumor thrombus

### Variceal bleeding and occurrence of rebleeding

Upper gastrointestinal tract endoscopic examination for the bleeding sites revealed that 58 (94 %) and 4 (6 %) patients had esophageal and gastric variceal bleeding, respectively. All were classified as GOV1. The bleeding was managed using EVL except for one patient who was treated with a Sengstaken-Blakemore tube. Complete initial hemostasis was achieved in all patients.

A total of 24 patients (39.3 %) experienced rebleeding. Although EVL successfully stopped the initial bleeding, 33 rebleeding events occurred in 62 patients (0–5 rebleeding events in each patient). A total of 4 and 14 patients experienced rebleeding within 7 and 30 days, respectively (Table 2).

**Table 2:** Outcomes of endoscopic variceal band ligation

Hemostasis rate	98.4 % (61/62)	An SB tube was used in one patient
Rebleeding rate	39.3 % (24/61)	4 patients within 7 days
		14 patients within 30 days
Occurrence of rebleeding (frequency)	33 events	
Median rebleeding-free survival time	29 days	
MST	36 days	

*MST* median survival time, *SB* Sengstaken-Blakemore

### Rebleeding-free and overall survival

**Fig. 2:**
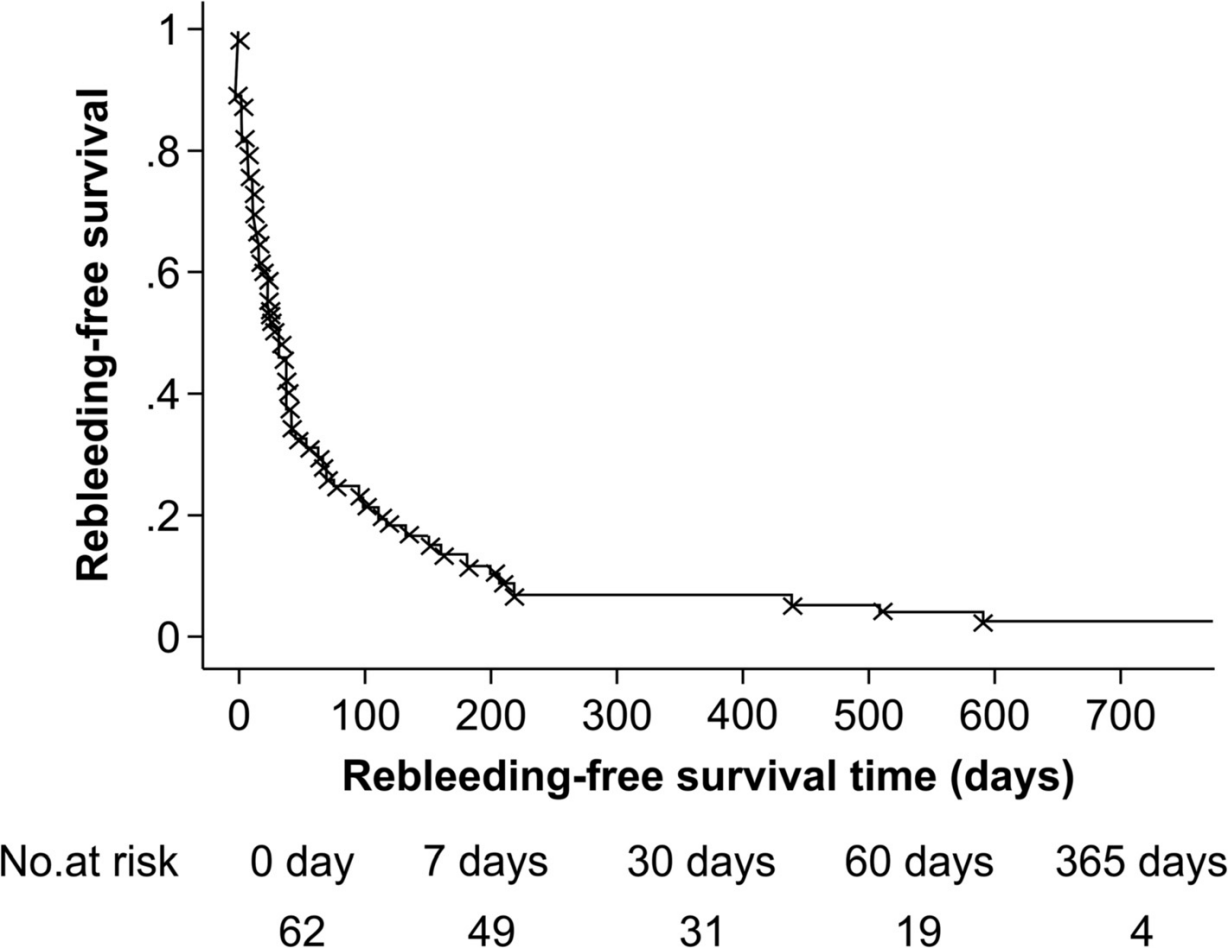
Rebleeding-free survival. The 7-, 30-, 60-day, and 1 year rebleeding-free survival rates were 79.0, 50.0, 30.6, and 6.5 %, respectively

The median rebleeding-free survival time was 29 days (95 % CI, 18–40 days), and the 7-, 30-, 60-day, and 1-year rebleeding-free survival rates were 79.0, 50.0, 30.6, and 6.5 %, respectively (Fig. 2).

**Fig. 3:**
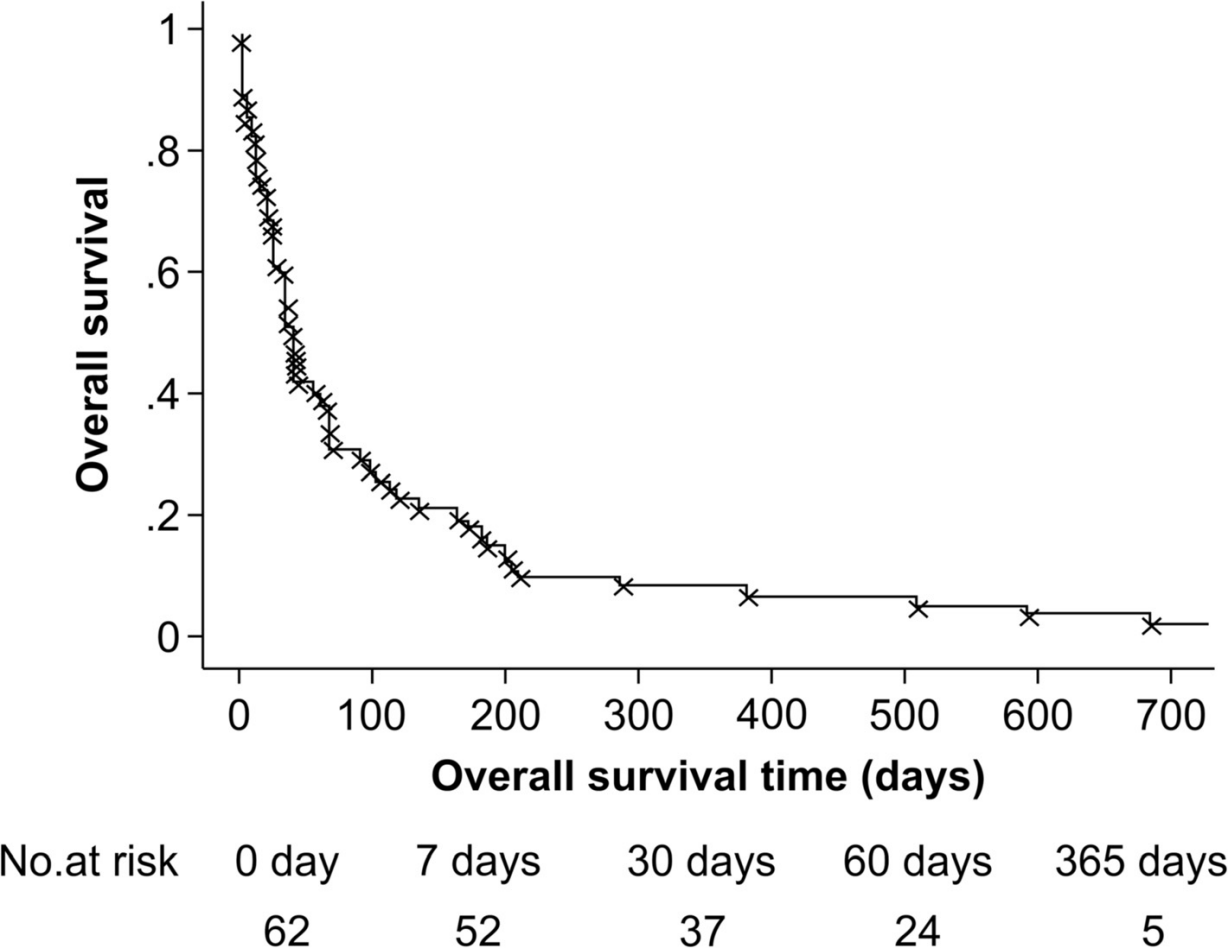
Overall survival. The 7-, 30-, 60-day, and 1-year overall survival rates were 83.9, 59.7, 38.7, and 8.1 %, respectively

**Fig. 4:**
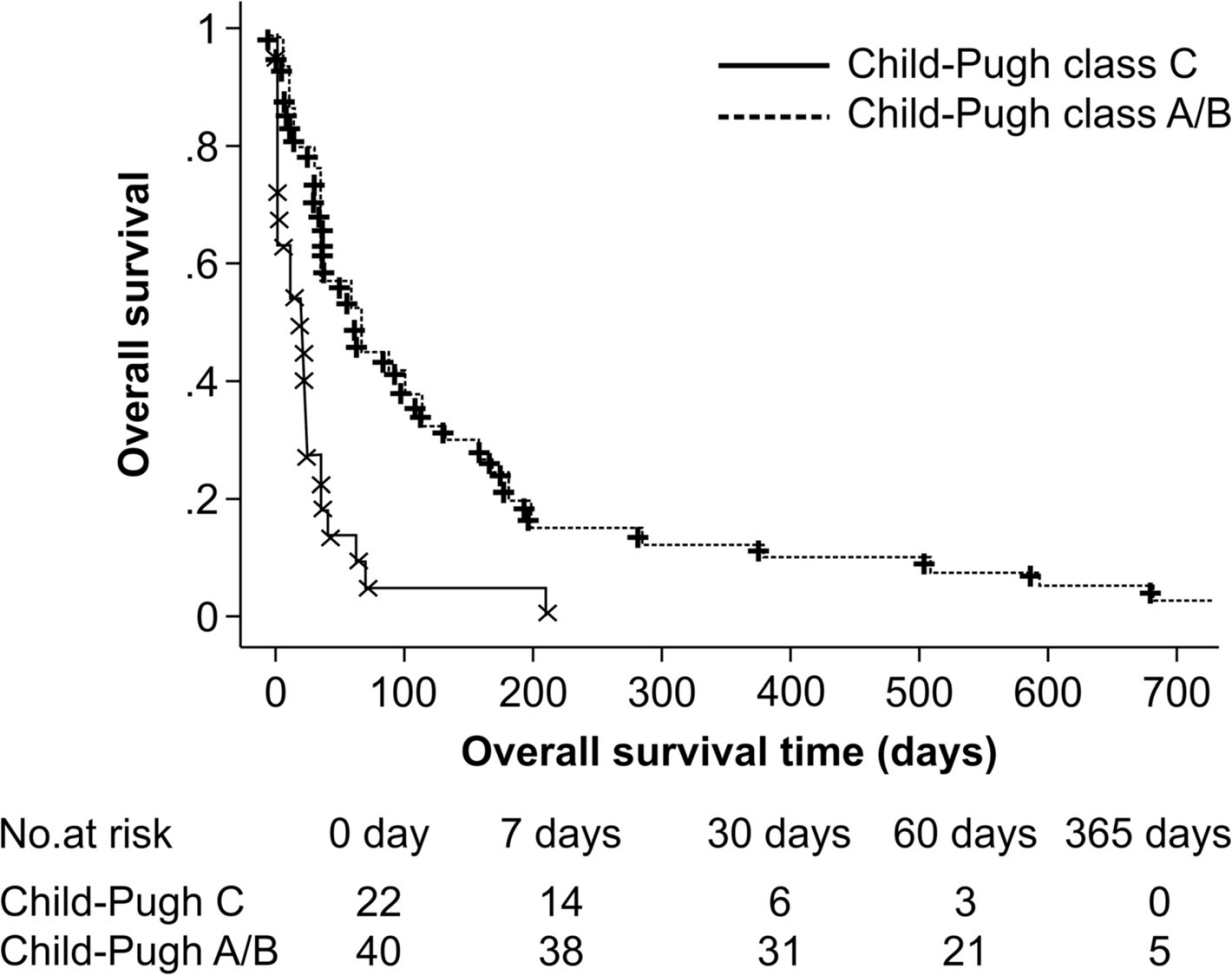
Overall survival based on Child-Pugh classification. The *solid line* connecting the “×” symbols indicates the overall survival of patients classified as Child-Pugh class C, whereas the *dotted line* connecting the “+” symbols indicates the overall survival of patients classified as Child-Pugh class A/B (*p* < .0001)

We detected a median overall survival time of 36 days (95 % CI, 23–63 days; Fig. 4). The 7-, 30-, 60-day, and 1-year overall survival rates were 83.9, 59.7, 38.7, and 8.1 %, respectively (Fig. 3). Despite achieving initial hemostasis, 10 patients died within 1 week. Only one patient survived for more than 5 years in this study. This patient developed HCC following hepatitis B-related cirrhosis in 2003. In 2007, a PVTT was found in the left branch of the patient’s portal vein, and arterial infusion chemotherapy with 5-fluorouracil in combination with interferon was administered [28]. An esophageal varix rupture occurred during chemotherapy, and EVL was performed. After hemostasis, arterial infusion chemotherapy was continued, resulting in tumor shrinkage and PVTT disappearance. The patient did not experience any rebleeding and was still alive in 2015.

### Prognostic factors for rebleeding-free survival

Among the 21 possible predictors we examined, our univariate analysis showed that a PVTT in the main trunk was the only significant factor affecting rebleeding-free survival (hazard ratio 3.706, *p* = 0.0223; Table 3). The median rebleeding-free survival time was 24 days in the patient group with a PVTT in the main portal trunk and 34 days in the patient group with a PVTT in the first or second branches of the portal vein (*p* = 0.6313).

**Table 3:** Significant predictors for rebleeding-free survival

Variable	Hazard ratio	95 % CI	*p* value
Age >62 years	0.466	0.165–1.318	0.1500
Sex (male)	1.129	0.292–4.360	0.8602
HCV	1.046	0.338–3.238	0.9382
NBNC	1.778	0.403–7.845	0.4474
Ascites (+)	1.333	0.354–5.026	0.6709
Encephalopathy (+)	1.780	0.382–7.559	0.4857
Albumin >2.9 g/dL	0.714	0.255–2.003	0.5224
Total bilirubin >1.4 mg/dL	0.436	0.153–1.244	0.1208
Prothrombin time >68 %	1.729	0.615–4.860	0.2987
Platelet count >12.0 × 10^4^/mm^3^	2.147	0.759–6.074	0.1500
AST >117 IU/L	0.643	0.229–1.804	0.4012
ALT >48.5 IU/L	1.729	0.615–4.860	0.2987
Child-Pugh class C	0.458	0.149–1.414	0.1746
Tumor size (>6.8 cm)	1.438	0.86–2.403	0.166
Tumor number (single)	0.287	0.031–2.662	0.2687
Extrahepatic metastasis (+)	1.153	0.369–3.600	0.8064
PVTT (main trunk)	3.706	1.205–11.401	0.0223
AFP >100 ng/dL	0.714	0.234–2.177	0.5540
AFP-L3 > 37.4 %	1.385	0.439–4.372	0.5790
DCP >100 ng/dL	3.485	0.381–31.839	0.2687
Variceal rupture (stomach)	5.286	0.516–54.103	0.1606

*AFP* alpha-fetoprotein, *AFP-L3* lectin-reactive alpha-fetoprotein, *ALT* aspartate aminotransferase, *AST* alanine aminotransferase, *CI* confidence interval, *DCP* des-gamma carboxyprothrombin, *HCV* hepatitis C virus, *NBNC* non-B non-C hepatitis, *PVTT* portal vein tumor thrombus

### Prognostic factors for overall survival

Sixteen in-hospital deaths occurred among the patients included in this study. Overall survival was affected by Child-Pugh class (*p* < 0.0001), AFP-L3 levels (*p* = 0.0080), and prothrombin time (*p* = 0.0161) in the univariate analysis (Table 4). The multivariate analysis showed that Child-Pugh class A/B (hazard ratio 0.398, *p* = 0.0065) and AFP-L3 levels <37.4 % (hazard ratio 0.464, *p* = 0.0147) were independent prognostic factors of overall survival (Table 5). The median overall survival time was 18 days for Child-Pugh class C patients and 64 days for Child-Pugh class A/B patients (*p* < 0.0001, Fig. 4).

**Table 4:** Independent prognostic factors for overall survival (univariate analysis)

Variable	Hazard ratio	95 % CI	*p* value
Age <62 years	0.752	0.448–1.263	0.2813
Sex (female)	0.794	0.412–1.529	0.4895
HBV	0.859	0.402–1.836	0.6956
HCV	1.509	0.727–3.131	0.2698
Ascites (−)	0.923	0.408–1.747	0.8058
Encephalopathy (−)	0.532	0.289–1.135	0.1425
Albumin <2.9 g/dL	1.084	0.651–1.805	0.7565
Total bilirubin <1.4 mg/dL	0.601	0.359–1.007	0.0533
Prothrombin time <68 %	1.882	1.125–3.150	0.0161
Platelet count <12.0 × 10^4^/mm^3^	0.692	0.413–1.158	0.1618
AST <117 IU/L	0.631	0.374–1.063	0.0834
ALT <48.5 IU/L	0.744	0.442–1.253	0.2664
Child-Pugh class A/B	0.350	0.179–0.554	<0.0001
Tumor size (<6.8 cm)	0.696	0.416–1.163	0.1540
Tumor number (multiple)	2.296	0.903–5.842	0.0810
Extrahepatic metastasis (−)	0.800	0.455–1.407	0.4385
PVTT (without main trunk)	1.284	0.762–2.162	0.3467
AFP <100 ng/dL	0.619	0.352–1.088	0.0958
AFP-L3 < 37.4 %	0.452	0.252–0.813	0.0080
DCP <100 ng/dL	0.481	0.189–1.220	0.1233
Variceal rupture (esophagus)	1.209	0.435–3.344	0.7193
EVL (−)	2.031	0.279–14.802	0.4844
Rebleeding (−)	1.091	0.651–1.826	0.7415

*AFP* α-fetoprotein, *AFP-L3* lectin-reactive α-fetoprotein, *ALT* aspartate aminotransferase, *AST* alanine aminotransferase, *CI* confidence interval, *DCP* des-gamma carboxyprothrombin, *EVL* endoscopic variceal band ligation, *HBV* hepatitis B virus, *HCV* hepatitis C virus, *PVTT* portal vein tumor thrombus

**Table 5:** Independent prognostic factors for overall survival (multivariate analysis)

Variable	Hazard ratio	95 % CI	*p* value
Prothrombin time <68 %	1.383	0.742–2.575	0.3071
Child-Pugh class A/B	0.398	0.205–0.773	0.0065
AFP-L3 < 37.4 %	0.464	0.251–0.860	0.0147

*AFP-L3* lectin-reactive α-fetoprotein, *CI* confidence interval

### Immediate death rates

We observed 95 bleeding events in 62 patients (1–6 events in each patient). EVL achieved hemostasis in 92 of the 95 bleeding events, and seven immediate deaths due to variceal bleeding (7/92; 7.6 %) were recorded. Of the three bleeding events treated with modalities other than EVL, all resulted in immediate deaths, indicating that EVL is useful for preventing immediate deaths after variceal bleeding (*p* = 0.0009).

## Discussion

Up to 40 % of patients with acute bleeding from esophageal varices have been reported to die from exsanguination; in other cases, death is due to bleeding complications, including liver failure, infections, and hepatorenal syndrome [29, 30]. Our study shows that EVL can produce a high rate of successful hemostasis in patients with variceal ruptures and a PVTT. EVL-treated bleeding events resulted in significantly lower immediate mortality rates than those treated with other modalities, suggesting that EVL is useful for preventing immediate death due to exsanguinating variceal bleeding.

Other than EVL, treatments for variceal bleeding include medical treatment with vasoactive drugs, balloon tamponade, placement of fully covered self-expanding metallic stents [31], transjugular intrahepatic portosystemic shunts [32], and surgical shunts [33]. Self-expanding metallic stents are not considered effective for the treatment of variceal bleeding due to their high associated incidence of rebleeding [34]. Transjugular intrahepatic portosystemic shunts and surgical shunts are also not indicated in patients with a PVTT and/or poor liver function. Some studies have reported endoscopic treatment of varices in patients with portal HCC invasion. Ng et al. reported data on 14 patients with bleeding esophageal varices and HCC, including 12 who had a PVTT. Successful bleeding control by emergency EIS was achieved for only 6 of 22 bleeding episodes (27 %) [35]. Nakashima et al. reported that in 6 patients with bleeding esophageal varices and HCC portal invasion, the initial variceal hemorrhage was successfully controlled following EIS (100 %). However, 4 of the 6 patients (66.7 %) experienced rebleeding and died of a hemorrhage and liver failure within 1 month [36]. In a study by Ohta et al., acute variceal bleeding was controlled following emergency EIS in 28 of 29 PVTT patients with bleeding esophageal varices (96.6 %) [37]. Our study showed a hemostasis rate of 98.3 %. Thus, based on these earlier reports, EVL was as effective as EIS. Moreover, EVL is now widely available, complications are less common than for EIS [18], and the associated mortality rates are lower than for sclerotherapy [14, 15]. Our findings confirm that EVL is effective for hemostasis and prevention of immediate death in patients with HCC and a PVTT.

In the present study, we did observe a high rate of rebleeding (39.3 %). A PVTT in the main trunk was predictive for rebleeding. In HCC patients with a PVTT in the main trunk, varices are exacerbated due to the marked elevation of portal vein pressure, leading to rebleeding despite initial hemostasis. The treatment of rebleeding is often difficult in these patients, and secondary prevention of variceal rupture is important for improving the patients’ prognoses.

We only studied the emergency treatment of variceal ruptures and therefore cannot discuss the advantages of preventive therapy for varices after initial hemostasis. However, variceal and concurrent HCC treatment might decrease bleeding frequency and improve patient prognosis. For example, Wu et al. reported that sorafenib, an oral multikinase inhibitor, could improve the outcome of variceal bleeding in patients with portal trunk or main branch HCC invasion [38]. In addition, Katamura et al. showed that intra-arterial 5-fluorouracil/interferon-α combined with three-dimensional conformal radiation therapy for a PVTT improved the PVTT response rate and reduced the incidence of portal hypertension-related events [39]. The median survival time of the patients classified as Child-Pugh class A was 133 days in this study, which might be long enough to effectively shrink a PVTT using chemotherapy with sorafenib, 5-fluorouracil hepatic arterial infusion with systemic interferon, or radiation to reduce portal vein pressure and the risk of rebleeding. Therefore, we speculate that despite being limited to patients in good general condition having good hepatic function, treatment for PVTT, leading to decreased portal vein pressure, might prevent rebleeding. In this study, one patient survived for more than 5 years. If endoscopic hemostasis is successful and liver cancer is controlled, as in this patient, prolonged survival of similar patients might be achievable.

After initial hemostasis success using EVL, rebleeding-free survival was predicted by the presence of a PVTT in the main trunk influencing portal vein pressure, and overall survival was predicted by Child-Pugh class and AFP-L3 levels, which are not directly related to variceal severity, but rather reflect liver function and the malignant potential of the HCC a determinant of prognosis. Thus, EVL treatment of bleeding varices appears to be sufficiently effective in ensuring that patient survival is no longer affected by bleeding varices but by overall liver function and tumor progression. For better survival of HCC patients with a PVTT concurrent with varices, control of HCC/PVTT and preservation or restoration of liver function are as important as variceal treatment.

Although our study showed that the prognosis of HCC patients with a PVTT and bleeding varices was still poor, preventative therapy for varices might prevent variceal ruptures. Since this study was a retrospective study dealing with only emergency hemostasis, the significance of prophylactic variceal treatment in HCC patients with a PVTT could not be assessed. A future prospective study on preventive variceal therapy in these patients is required.

## Conclusions

We evaluated the long- and short-term outcomes of patients with a PVTT who experienced potentially fatal ruptures of their esophageal or gastric varices. We found a rebleeding rate of 39.3 % and a median overall survival time of 36 days. A PVTT in the main portal trunk was predictive of rebleeding, and AFP-L3 levels and Child-Pugh class were predictive of overall survival. All EVL-treated patients in this study achieved initial complete hemostasis. Therefore, EVL of variceal ruptures in patients with a PVTT can achieve hemostasis and is useful for preventing immediate death from variceal bleeding. We conclude that EVL is a safe and effective treatment of variceal ruptures in patients with HCC and PVTT. After successful hemostasis, the alleviation of the underlying liver function impairment and tumor control are equally important for a good prognosis.

### Ethics statement

All procedures were in accordance with the ethical standards of the responsible committee on human experimentation (institutional and national) and with the Helsinki Declaration of 1975, as revised in 2008 [5]. This study was approved by the Kyoundo Hospital’s ethics committee, and written informed consent was obtained from all patients.

### Availability of data and materials

All the data supporting the study findings are presented in the main manuscript.

## Abbreviations

CT: computed tomography
EIS: endoscopic injection sclerotherapy
EVL: endoscopic variceal band ligation
GOV: gastroesophageal varices
HCC: hepatocellular carcinoma
IGV: isolated gastric varices
PVTT: portal vein tumor thrombus.
